# Functional Genetic Variants of *TNFSF15* and Their Association with Gastric Adenocarcinoma: A Case-Control Study

**DOI:** 10.1371/journal.pone.0108321

**Published:** 2014-09-24

**Authors:** Zhi Zhang, Dianke Yu, Jie Lu, Kan Zhai, Lei Cao, Juan Rao, Yingwen Liu, Xuemei Zhang, Yongli Guo

**Affiliations:** 1 Department of Chemotherapy and Radiotherapy, Tangshan Gongren Hospital, Tangshan, China; 2 Institute of Molecular Genetics, College of Life Science, Hebei United University, Tangshan, China; 3 Department of Etiology of Carcinogenesis, Cancer Institute, Chinese Academy of Medical Sciences and Peking Union Medical College, Beijing, China; 4 Beijing Key Laboratory for Pediatric Otolaryngology, Head and Neck Science, Beijing Pediatric Research Institute, Beijing Children’s Hospital, Capital Medical University, Beijing, China; MOE Key Laboratory of Environment and Health, School of Public Health, Tongji Medical College, Huazhong University of Science and Technology, China

## Abstract

The purpose of this study was to identify functional genetic variants in the promoter of tumor necrosis factor superfamily member 15 (*TNFSF15*) and evaluate their effects on the risk of developing gastric adenocarcinoma. Forty DNA samples from healthy volunteers were sequenced to identify single nucleotide polymorphisms (SNPs) in the *TNFSF15* promoter. Two *TNFSF15* SNPs (−358T>C and −638A>G) were identified by direct sequencing. Next, genotypes and haplotypes of 470 gastric adenocarcinoma patients and 470 cancer-free controls were analyzed. Odds ratios (ORs) and 95% confidence intervals (CIs) were estimated by logistic regression. Serologic tests for *Helicobacter pylori* infection were measured by enzyme-linked immuno-sorbent assay (ELISA). Subjects carrying the *TNFSF15* −358CC genotype were at an elevated risk for developing gastric adenocarcinoma, compared with those with the −358TT genotype (OR 1.42, 95% CI, 1.10 to 2.03). *H. pylori* infection was a risk factor for developing gastric adenocarcinoma (OR 2.31, 95% CI, 1.76 to 3.04). In the *H. pylori* infected group, subjects with *TNFSF15* −358CC genotype were at higher risks for gastric adenocarcinoma compared with those carrying −358TT genotype (OR: 2.01, 95%CI: 1.65 to 4.25), indicating that *H. pylori* infection further influenced gastric adenocarcinoma susceptibility. The −358 T>C polymorphism eliminates a nuclear factor Y (NF-Y) binding site and the −358C containing haplotypes showed significantly decreased luciferase expression compared with −358T containing haplotypes. Collectively these findings indicate that functional genetic variants in *TNFSF15* may play a role in increasing susceptibility to gastric adenocarcinoma.

## Introduction

Gastric cancer is one of the most common cancers worldwide. According to the GLOBOCAN project, 952 000 new gastric cancer cases were estimated to have occurred in 2012, which attributed to 6.8% of newly diagnosed cancers [1]. *Helicobacter pylori* (*H. pylori*), a gram-negative bacterium, is one of the most important environmental risk factors for gastric cancer [2]–[4]. However, the mechanism underlying the development and progression of gastric cancer influenced by *H. pylori* infection is still unclear.

It has been suggested that inflammation is an important mediator of gastric cancer induced by *H. Pylori* and tumor necrosis factors (TNFs) is a key regulator of inflammation and has been indicated as a contributing factor for the development and progression of tumors [5]–[8]. Tumor necrosis factor superfamily member 15 (TNFSF15), also known as vascular endothelial growth inhibitor (VEGI) or TNF ligand related molecule 1 (TL1), is a unique cytokine that functions as a modulator of vascular homeostasis and inflammation [9]–[12]. The *TNFSF15* gene is located on chromosome 9q32, and the full length of this gene is approximately 17 kb. TNFSF15 shares 20–30% identity (or similarity) in amino acid sequence with other TNF family members and has similar functions [13]–[15]. TNFSF15 is involved in numerous cellular processes including the suppression of neovascularization which is essential for tumor progression and spread [12], [16], [17]. Over-expression of *TNFSF15* gene has been shown to inhibit the development of multiple tumor types [18], [19]. The TNFSF15 protein also inhibits the tumor growth in murine tumor models [13], [20]. Recently, TNFSF15 was also recognized as a valuable potential therapeutic target for cancer therapy [21], [22]. Collectively these findings suggest an important role of TNFSF15 in cancer suppression.

*TNFSF15* gene is polymorphic, and single nucleotide polymorphisms (SNPs) in this gene were identified by genome-wide association study (GWAS) in Japanese cohort [23], but the frequency of these SNPs in other populations still have to be examined. Among these SNPs, several genetic variants in the promoter region of *TNF15* have been documented to associate with increased risk to Crohn’s Disease in Japanese and European cohort, while the biological function was not reported [23], [24]. Moreover, little or nothing is known about the effects of these *TNFSF15* polymorphisms on human cancer susceptibility.

In view of the importance of TNFSF15 in tumor progression and metastasis, we hypothesized that the *TNFSF15* SNPs in promoter region may influence protein expression and function, which confers susceptibility to gastric cancer. Therefore, in this study we sought to identify the functional polymorphisms in the promoter of *TNFSF15* gene and conduct a case-control study to investigate the frequency of these SNPs and the possible association with the risk for developing gastric cancer in the Chinese population.

## Methods

### *TNFSF15* SNPs identification

Forty DNA samples derived from peripheral blood of unrelated healthy Han Chinese individuals were used to search for SNPs within the promoter of *TNFSF15* (1274 bp). The PCR primers were as follows: Primer F (5′-TCC AAC ACC ACC TCT TTC TCC AG −3′) and Primer R (5′-TAT GTG GTG AGT CCT GCA AGG −3′). PCR products were bidirectionally sequenced to identify the genetic variants in the *TNFSF15* promoter (373 Automated DNA Sequencer, Applied Biosystems, Foster City, USA). Finally, we used the Mutation Explorer program (Todaysoft Inc, Beijing, China) to identify SNP candidates that were further confirmed by re-amplifying and re-sequencing SNP sites from the opposite DNA strand.

By re-sequencing the *TNFSF15* promoter of 40 healthy subjects, we identified two genetic variants (−358 T>C, rs6478109 and −638 A>G, rs7848647), which were located at −358 bp and −638 bp upstream of the translation start site, ATG.

### Study participants

This hospital-based, case-control study consisted of 470 patients with histopathologically verified primary gastric adenocarcinoma and 470 healthy individuals. All subjects were unrelated and ethnically classified as Han Chinese. The cases were consecutively recruited (90% recruitment rate) from January 1997 to January 2004, at the Cancer Hospital, Chinese Academy of Medical Sciences (Beijing). The exclusion criteria included previous cancer and previous chemotherapy or radiotherapy. The stage was evaluated according to the UICC Tumor-Node-Metastasis (TNM) classification for gastric carcinoma at diagnosis on the basis of postoperative pathological examination of specimens. The control subjects were randomly selected from a database consisting of 2500 individuals based on a physical examination. The selection criteria included no history of cancer and matched the frequencies of study cases for age and sex. The detailed recruitment of patients and controls was described previously [25]. At recruitment, written informed consent was obtained from each subject and this study was approved by the Institutional Review Board of the Cancer Institute, Chinese Academy of Medical Sciences.

### *TNFSF15* Genotyping Assays

Genotypes were determined by PCR-based restriction fragment length polymorphism (PCR-RFLP), using −358F(5′-AAA TGT GAT TTC CGT TTC CCC A -3′)/−358R (5′-TGG GTG GGG CAA AAT ATA CC-3′) and −638F (5′-AGT CAC CTC GAT CTG TGG CCT C-3′)/−638R (5′-AAT CAC GGC TTG GAG TTG TAA CCT C-3′) as PCR primer sets for −358T>C (rs6478109) or −638A>G (rs7848647) sites, respectively. The PCR profile consisted of an initial denaturation for 2 min at 95°C, followed by 35 cycles of 25 s at 94°C, 25 s at 59°C, 25 s at 72°C, and a final elongation step of 5 min at 72°C. The PCR products were then digested with *Bcc*I (for −358 T>C site) or *Rsa*I (for −638 T>C site) (New England BioLabs, Inc., Beverly, USA) and separated on a 3% agarose gel. *TNFSF15* genotypes detected by PCR-RFLP analysis were further confirmed by DNA sequencing. Genotyping was performed without knowledge of subjects’ case/control status. To ensure the quality control, 10% random samples of cases and controls were tested twice by different persons and results were in 100% concordance.

### Serologic Tests for *H. Pylori* Infection

Serologic assay was performed for the *H. pylori* infection status as previously described [26]. In brief, *H. pylori* stains cultured from gastric biopsies of five patients in a former study were used to provide a local antigen preparation for serology. Serum levels of anti- *H. pylori* IgG and IgA were measured separately in duplicate with ELISA procedures. Quality-control samples were assayed at Vanderbit University, Nashville, Tennessee. An individual was determined to be positive for *H. pylori* infection if the mean optical density for either anti- *H. pylori* IgG or anti- *H. pylori* IgA was >0.1, a cut-off value from the examination of a group of *H. pylori* -negative persons and reference sera. Serum levels of sCD14 were measured by commercially available ELISA kits (JingMei Biotech, Shenzhen, China). The concentration of each sample (unknown) was determined by extrapolation from a standard curve estimated from a panel of standards of known concentrations.

### Plasmid Construction

A pGL3-basic reporter plasmid encompassing −1079/−1 bp of *TNFSF15* was constructed, using 5′-ATG CGG TAC CAG AAG CCA GCA GCC AGC CT-3′ and 5′-ATG CGC TAG CGC TCC TGC TGC TCC TGG AGG -3′ as PCR primers. The resulting construct was named as pA_−638_-C_−358_ according to sequence analysis. Subsequently, site-specific mutagenesis was performed to generate other constructs, pA_−638_-T_−358_, pG_−638_-C_−358_, and pG_−638_-T_−358_, by using pA_−638_-C_−358_ as templates. All constructs used in this study were restriction mapped and sequenced to confirm their authenticity.

### Luciferase Reporter Gene Assays

Human gastric carcinoma cell lines (MGC-803, AGS, and BGC-823), originally purchased from Cell Resource Center, Chinese Academy of Medical Sciences (CAMS, Beijing, China), were cultured in RPMI 1640 (Gibco, Grand Island, USA) (for MGC-803 and AGS cells) or F12 medium (Gibco, Grand Island, USA) (for BGC-823 cells) supplemented with 10% fetal bovine serum, in a humidified, 5% CO_2_ incubator at 37°C. Next, 1×10^4^ cells were plated in 48-multiwell plates (Corning, NY, USA) and grown to 80–90% confluence for transient transfection using Lipofectamine 2000 Reagent (Life Technologies, Inc., Rockville, USA) according to the manufacturer’s protocol. Cells were then co-transfected with 500 ng of pA_−638_-C_−358_, pA_−638_-T_−358_, pG_−638_-C_−358_, and pG_−638_-T_−358_, or PGL3-basic plasmids and 1.0 ng of *Renilla* luciferase reporter plasmid pRL-SV40 (Luciferase Assay System, Promega, Madison, USA) for standardization of the transfection efficiencies. Luciferase activity was measured using the Dual-Luciferase Reporter Assay system (Promega, Madison, USA) on a TD-20/20^n^ Luminometer (Turner Designs, Promega, Madison, USA) according to the manufacturer’s protocol. Results were normalized for *Renilla* activity and were expressed as relative luciferase activity (RLA). Three independent transfection experiments were performed, and each was done in triplicate.

### Electrophoretic Mobility Shift Assay (EMSA)

Synthetic biotin labeled double-stranded oligonucleotides were −358T probe (5′-biotin-ATT TCC GTT TCC CAA TCT GCA AAC CAC ACA-3′/5′-biotin-TGT GTG GTT TGC AGA TTG GGA AAC GGA AAT-3′) and −358C probe (5′-biotin-ATT TCC GTT TCC CAA CCT GCA AAC CAC ACA-3′/5′-biotin-TGT GTG GTT TGC AGG TTG GGA AAC GGA AAT-3′). The gel shift assay was accomplished using the LightShift Chemiluminescent EMSA Kit (Pierce, Rockford, IL, USA) according to the manufacturer’s instructions. For each gel shift reaction, an aliquot of labeled oligonucleotide (10 fmoles) was incubated with 3 µg of nuclear extract from HeLa cells (Promega). For competition experiments, a 100-fold molar excess of unlabeled −358T or −358C oligonucleotide, a NF-Y recognition element (NF-Y cons, 5′-AGT TCA TCA GCC AAT CAG AGC ACA GG-3′/5′-CCT GTG CTC TGA TTG GCT GAT GAA CT-3′), or a mutant NF-Y recognition element (NF-Y mut, 5′-AGC TGT AGA GTG TGA GTG ATG AAG ACA T-3′/5′-ATG TCT TCA TCA CTC ACA CTC TAC AGC T-3′) was pre-incubated for 2 minutes at room temperature with the nuclear extracts before the addition of the labeled probe. Samples were then run on a non-denaturing 6% polyacrylamide gel and the electrophoresed binding reactions were transferred to positively charged nylon membranes (Pierce) in a Mini Trans-Blot Cell (BioRad, Hercules, USA). Cross-link was performed in a GS Gene Linker UV chamber (BioRad, Hercules, USA). Detection of biotin-labeled DNA was performed using stabilized streptavidin/horseradish peroxidase conjugate (Pierce) according to the manufacturer’s instructions.

### Haplotype Construction and Statistical Analysis

Unconditional logistic regression was used to assess the association between genotypes and risk of gastric cancer using the Statistical Analysis System software (version 6.12; SAS Institute, Cary, USA). Odds ratios (ORs) were adjusted for age and sex where it was appropriate. HaploStats software package (Mayo Clinic/Foundation, Rochester, USA, http://mayoresearch.mayo.edu/mayo/research/schaid_lab/software.cfm) developed using the R language was employed to estimate and test haplotype-environment interactions in the general linear model framework. Simulations were run 1000 times for empirical *P*-values. Haploview 3.2 software (http://www.broad.mit.edu/mpg/haploview/) was also used to construct the haplotypes, evaluate the linkage disequilibrium of two SNPs and test the association of single markers and haplotypes with gastric carcinomas, correcting for multiple testing bias by permutation tests.

### Bioinformatics

The genetic variants identified were compared with the Ensemble database (ENSG00000181634, http://asia.ensembl.org). The potential transcription factor binding capability between two different alleles of TNFSF15 −358 T>C and −638 A>G were predicted by TRANSFAC program and the accompanying MATCH at http://www.gene-regulation.com.

## Results

### Identification of SNPs in the *TNFSF15* promoter

By resequencing the *TNFSF15* promoter of 40 healthy subjects, we identified two genetic variants (−358 T>C, rs6478109 and −638 A>G, rs7848647), which were located at −358 bp and −638 bp upstream of the translation start site, ATG. The allele frequencies for the −358C and −638G were 0.498 and 0.485 in healthy controls, respectively. No novel SNP were identified.

Next, we estimated the degree of linkage disequilibrium between the two SNPs, using two marker expectation maximizations (EMs) to estimate the maximum-likelihood values of the four-gamete frequencies. Results indicated that these two polymorphisms were in linkage disequilibrium, with the *D*′ = 0.932, LOD = 142.15, r^2^ = 0.826 in our study population.

### Subject Characteristics

As shown in Table 1, no statistically significant difference was found between 470 cases and 470 controls in terms of age (*P* = 0.553) and sex (*P* = 0.385). However, the percentage of *H.* *pylori* infection in patients was higher than in controls (73.2% versus 54.3%). Among 470 patients, 383 (81.5%) had detailed tumor stage information, while this information was unavailable for the remaining patients (18.5%).

**Table 1:** Distribution of selected characteristics of gastric cancer patients (cases) and controls.

	Cases (n = 470)		Controls (n = 470)		
	No.	(%)	No.	(%)	*P-*value*
Sex					0.385
Male	330	(70.2)	342	(72.8)	
Female	140	(29.8)	128	(27.2)	
Age (years)					0.553
≤57	253	(53.8)	242	(51.5)	
>57	217	(46.2)	228	(48.5)	
*H pyroli* infection					<0.0001
Negative	126	(26.8)	215	(45.7)	
Positive	344	(73.2)	255	(54.3)	
Tumor stage†					
0	6	(1.3)			
I	69	(14.7)			
II	63	(13.4)			
III	117	(24.8)			
IV	128	(27.2)			
Unknown	87	(18.5)			

*Two-sided χ^2^ test.

† According to the UICC Tumor-Node-Metastasis classification for gastric carcinoma (1997).

### Association of *TNFSF15* Promoter Polymorphisms with Gastric Adenocarcinoma

The frequencies of *TNFSF15* −358 T>C and −638 A>G genotypes in cases and controls and their association with gastric adenocarcinoma are shown in Table 2. The observed genotype distribution of −358 T>C and −638 A>G polymorphisms within the control population did not deviate significantly from those expected from the Hardy-Weinberg equilibrium (*P* = 0.871 and 0.715, respectively). The frequencies of *TNFSF15* −358 T>C genotypes in infected cases (TT, 22.8%; TC, 45.5%; CC, 31.7%) were significantly different from those in controls (TT, 24.9%; CT, 50.6%; CC, 24.5%) (*P* = 0.047). Logistic regression analysis showed an elevated risk of gastric adenocarcinoma for subjects with *TNFSF15* −358CC genotype (OR 1.42, 95% CI, 1.10 to 2.03, *P* = 0.04), but not for subjects carrying *TNFSF15* TC genotype (OR 1.00, 95% CI, 0.72 to 1.40, *P* = 0.89), compared with *TNFSF15* −358TT genotype carriers. No significant difference between the genotype frequencies of *TNFSF15* −638 A>G polymorphism in cases and controls was observed (*P* = 0.216). In the stratification analysis, no significant differences in distributions of genotype frequencies were found among different stages of tumors.

**Table 2:** Genotype frequencies of *TNFSF15* polymorphisms among gastric cancer patients and controls and their association with the risk of gastric carcinoma.

Genotype	Patients (n = 470)		Controls (n = 470)		OR (95% CI)*	*P-*value
	No.	(%)	No.	(%)		
−358 T/C						
TT	107	(22.8)	117	(24.9)	1.00 (Reference)	
TC	214	(45.5)	238	(50.6)	1.00 (0.72–1.40)	0.89
CC	149	(31.7)	115	(24.5)	1.42 (1.10–2.03)	0.04
−638 A/G						
AA	118	(25.1)	122	(26.0)	1.00 (Reference)	
AG	221	(47.0)	240	(51.0)	0.94 (0.69–1.30)	0.729
GG	131	(27.9)	108	(23.0)	1.24(0.86–1.78)	0.256

*Adjusted for age, sex and *H.pylori* infection.

The haplotype frequencies computed using the HaploStats software are presented in Table 3. No significant difference in haplotype frequencies was observed between gastric adenocarcinoma patients and controls (χ^2^ = 5.65, *P = *0.130, *df* = 3).

**Table 3:** Risk estimates for extended *TNFSF15* haplotypes in gastric cancer patients and controls.

Haplotype	Patients (n = 470)	Controls (n = 470)	OR (95% CI)*	*P-*value
	No. of chromosomes (%)	No. of chromosomes (%)		
A_−638_–T_−358_	416 (44.3)	456 (48.5)	1.00 (Reference)	
G_−638_–T_−358_	471 (50.1)	441 (46.9)	1.17 (0.97–1.40)	0.123
A_−638_–C_−358_	40 (4.3)	27 (2.9)	1.48 (0.94–2.36)	0.099
G_−638_–C_−358_	13 (1.3)	16 (1.7)	0.83 (0.41–1.71)	0.564

*Adjusted for age, sex, and *H. pylori* infection.

### The interaction of TNFSF15 polymorphisms with *H. pylori* infection and gastric adenocarcinoma

The percentage of *H. pylori* infection was significantly higher in cases than in controls (73.2% versus 54.3%, *P*<0.0001). Logistic regression analysis showed that subjects with *H. pylori* infection were at a 2-fold higher risk for developing gastric adenocarcinoma compared to those without *H. pylori* infection (OR 2.31, 95% CI, 1.76 to 3.04, *P*<0.0001). Consequently, the risk of gastric adenocarcinoma associated with the *TNFSF15* genotype was further examined by stratifying gastric cancer patients by *H. pylori* infection status. Logistic regression analysis (Table 4) showed that the subjects with *TNFSF15* −358 CC genotype were at elevated risks for developing gastric adenocarcinoma compared with those with −358 TT genotype in the *H. pylori* infected group (OR 2.01, 95%CI, 1.65 to 4.25), but not in the *H. pylori* negative group (OR 0.87, 95% CI, 0.45 to 1.69). In addition, we have examined the potential effect of the −638 A>G variants interacting with *H. pylori* infection on the susceptibility to develop a gastric adenocarcinoma; however, no significant difference among different genotypes was observed.

**Table 4:** Risk of gastric carcinoma associated with *TNFSF15* genotypes by *H. pylori* infection status.

Genotype	*H. pylori* infection	Patients (n = 470)	Controls (n = 470)	OR (95% CI )*	*P-*value
		No. (%)	No. (%)		
−358 T/C					
TT	–	33 (7.0)	48 (10.2)	1.00 (Reference)	
TC	–	61 (13.0)	110 (23.4)	0.83 (0.46–1.49)	0.51
CC	–	32 (6.8)	57 (12.1)	0.87 (0.45–1.69)	0.65
TT	+	74 (15.7)	69 (14.7)	1.00 (Reference)	
TC	+	153 (32.6)	128 (27.2)	1.15 (0.75–1.85)	0.62
CC	+	117 (24.9)	58 (12.3)	2.01 (1.65–4.25)	0.006
–638 A/G					
AA	–	35 (7.4)	53 (11.3)	1.00 (Reference)	
AG	–	61 (13.0)	110 (23.4)	0.86 (0.50–1.46)	0.568
GG	–	30(6.4)	52 (11.1)	0.92 (0.67–1.26)	0.584
AA	+	83 (17.7)	69 (14.7)	1.00 (Reference)	
AG	+	160 (34.0)	130 (27.7)	1.04 (0.70–1.54)	0.859
GG	+	101 (21.5)	56 (11.9)	1.25 (0.99–1.57)	0.06

*Adjusted for age and sex.

### Different transcriptional activity of *TNFSF15* −358 T>C, −638 A>G haplotype

**Figure 1:**
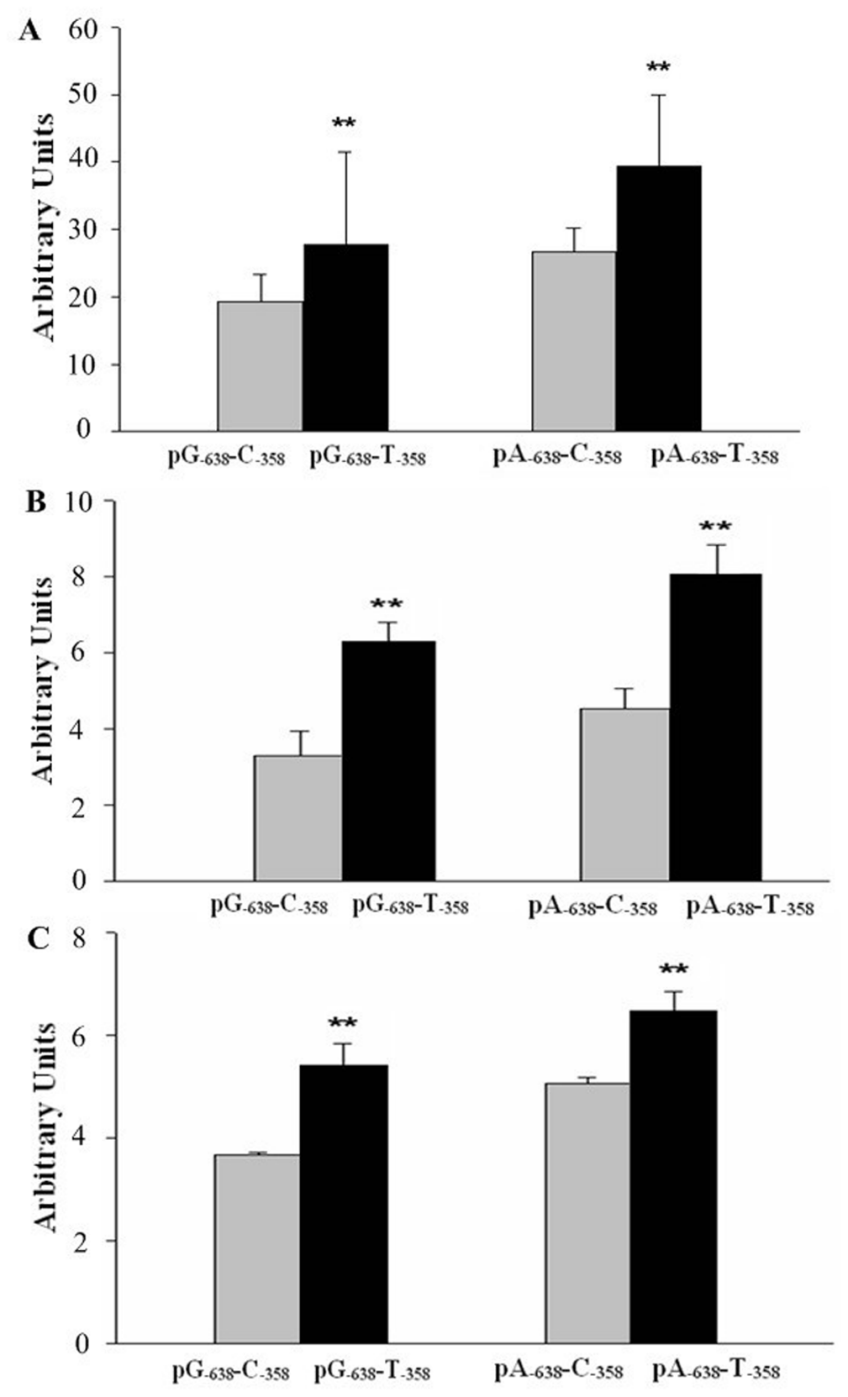
Transient reporter gene expression assays with constructs containing full-length TNFSF15 promoter. Luciferase expression of constructs in (A) MGC-803 cells, (B) AGS cells, and (C) BGC-823 cells. Luciferase levels were normalized to the results obtained for empty vector and shown as the means and standard deviation (S. D.) of fold increase from 3 independent transfection experiments, each performed in triplicate. **P<0.01 compared with the construct counterparts.

Since a linkage disequilibrium between −358 T>C and −638 A>G SNPs was observed, functional evaluation of the haplotype was performed. The transcriptional activity of four haplotypes generated by two *TNFSF15* −358 T>C and −638 A>G genetic variants were compared by transiently transfecting the pA_−638_-C_−358_, pA_−638_-T_−358_, pG_−638_-C_−358_, and pG_−638_-T_−358_ luciferase reporter constructs into human gastric cell lines MGC-803, AGS and BGC-823. As shown in Figure 1, all constructs containing the −358C allele consistently had lower expression levels of luciferase compared with constructs containing the −358T allele in MGC-803, AGS and BGC-823 cells. Reporter gene expression driven by the constructs with the −638A allele seemed to be higher than the one driven by the constructs with the −638G allele; however, the difference was not statistically significant (*P*>0.05). Taken together, these data support the influence of *TNFSF15* −358 T>C variant on *TNFSF15* promoter activity.

### Allelic-specific Binding of Nuclear Proteins to *TNFSF15* promoter

**Figure 2:**
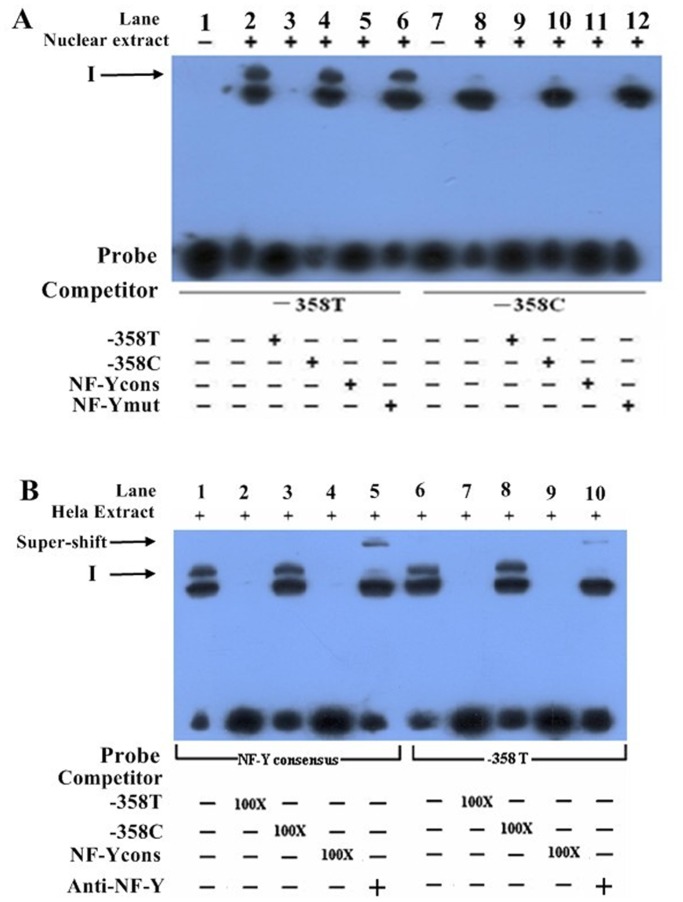
EMSA with −358T or −358C containing oligonucleotides allele and nuclear extracts from HeLa cells. (A) Nuclear protein extracts from HeLa cells were incubated with biotin-labeled oligonucleotide probes containing −358T or −358C. The figure shows mobility of the labeled oligonucleotides without nuclear extracts (lanes 1 and 7), with nuclear extracts in the absence of competitor (lanes 2 and 8), with nuclear extracts in the presence of various unlabeled competitors as indicated at the bottom autoradiograph (lanes 3–6 and 9–12). The arrow indicates a major oligonucleotide-nuclear protein complex. (B) Nuclear protein extracts from HeLa cells were incubated with biotin-labeled oligonucleotide probes containing −358T or consensus NF-Y binding element. The lower arrow indicates major oligonucleotide-nuclear protein complex without antibody to NF-Y, and the upper arrow shows the protein complex with antibody to NF-Y (lane 5 and 10).

Bioinformatics analysis showed that the *TNFSF15* −358T>C variant is located within a consensus sequence of the NF-Y binding site, and the T to C transition appears to disrupt NF-Y binding, and this could explain the lower promoter activity. For the −638A>G variant, the TRANSFAC program predicted no change in the binding sites of the transcriptional factors. Furthermore, electrophoretic mobility shift assay was performed to investigate whether the differences in *TNFSF15* promoter activity between the −358T and −358C alleles were due to their transcription factor binding activities. Nuclear protein extracts from HeLa cells were incubated with biotin-labeled oligonucleotide probes containing −358T or −358C alleles. As shown in the Figure 2A, a clear DNA-protein complex was detected with −358T probe (lane 2) but not with −358C probe (lane 8). Next, competition experiments were performed in order to determine the sequence specificity of this DNA-protein complex. The band was competed by 100-fold molar excess of unlabeled −358T probe (lane 3) but not by the same concentration of unlabeled −358C probe (lane 4). More so, this DNA-protein complex was completely inhibited by 100-fold molar excess of unlabeled NF-Y recognition element (lane 5) but not by the unlabeled mutated NF-Y recognition element (lane 6). Collectively these results show that −358T allele, but not the −358C allele, is able to bind specifically to the nuclear protein.

To verify that the nuclear protein is indeed NF-Y, the super-EMSA assay was performed using antibodies against NF-Y, in which nuclear protein extracts from HeLa cells were incubated with biotin-labeled oligonucleotide probes containing −358T or a consensus NF-Y binding element. As shown in Figure 2B, when the anti-NF-Y antibody was added, the super shift band was observed (lanes 5 and 10). These findings suggest that NF-Y may possibly bind to the promoter region of *TNFSF15 in vivo*.

## Discussion

Gastric cancer is one of the major health problems worldwide and genetic events underlying its initiation and progression still aren’t completely understood. In the present study, we identified two genetic variants (−358T>C and −638A>G) in the promoter of *TNFSF15* gene and examined their influence on *TNFSF15* transcriptional activity and the susceptibility to gastric adenocarcinoma in Chinese populations. In our study, subjects with −358CC genotype had a 1.42-fold increased risk for developing gastric cancer compared with −358TT genotype carriers. However, there was no significant difference in *TNFSF15* −638A>G genotype frequencies between cases and controls. These data indicated that the TNFSF15 −358T>C polymorphism might be involved in the development of gastric cancer.

Recent studies point to a possible anti-tumor role of TNFSF15. For instance, it has been shown that recombinant TNFSF15 exhibits anti-neoplastic effects by inhibiting angiogenesis and TNFSF15 overexpression was associated with improved cancer prognosis [20], [22], [27]. Moreover, TNFSF15 can suppress endothelial cell proliferation and induce apoptosis in endothelial cells via different kinase pathways [11], [28], [29].

To the best of our knowledge, this is the first study to evaluate the association of functional *TNFSF15* polymorphisms with susceptibility to cancer. Nevertheless, several studies have examined the relationship between *TNFSF15* polymorphisms and susceptibility to inflammatory bowel disease and Crohn’s disease in Japanese and European populations [23], [24], [30]. In our study, we have sequenced the full-length of *TNFSF15* promoter in a subset of 40 healthy Han Chinese subjects, and we have identified two polymorphisms (−358T>C and −638A>G). The −358C allele frequency in our control subjects was 0.498, which differs from that reported via GWAS in Japanese population (−358C: 0.593). The −638G allele frequency in our control population (−638G: 0.485) differed from that observed in Japanese (−638G: 0.593) and European populations (−638G: 0.655) as well [23], [24]. These differences might be due to relatively small sample size in our study and different ethnic backgrounds of the study participants. In addition, the allele frequencies of the 2 SNPs in this cohort were different to those listed in 1000 Genome project (−358C: 0.278 and −638G: 0.279). Additional studies based on larger sample sizes are needed to further verify these frequencies in different populations. Since genetic variants may have different influences on TNFSF15 expression, that ethnic differences in the distribution of *TNFSF15* genetic variants might partially correlate with their different effects on cancer phenotypes within these populations.

The function of promoter motifs and transcriptional factors in the regulation of TNFSF15 expression are still not fully elucidated. In our study, the *in silico* analysis of the −638A>G change in the *TNFSF15* promoter conferred neither gain nor loss of binding activities for any transcription factor. However, the change of nuclear transcription factor NF-Y binding activity was observed for the *TNFSF15* −358T>C promoter polymorphism with EMSA experiments, indicating NF-Y might play important roles in TNFSF15 expression. NF-Y is the CCAAT box activator which is one of the most frequent promoter elements [31], [32]. NF-Y has widespread activity, and is specifically required for genes regulated during the cell-cycle which is important for many critical cellular and developmental events including cancer progression [31]. Studies showed that inactivation of NF-Y gene in mouse model is lethal at early stage of development [33]. Furthermore, our reporter constructs encompassing the *TNFSF15* −358T allele showed stronger transcription activity than the constructs encompassing the −358C allele. These findings are in line with the results of our gastric cancer association study in which the −358CC genotype was associated with higher susceptibility to gastric cancers, indicating that the −358C allele might be a cancer-risk allele.

Taken together, these results point to the possible influence of *TNFSF15* −358T>C promoter polymorphism in the regulation of TNFSF15 expression in gastric tissue. In this case, it would be expected that *TNFSF15* genetic variants might contribute to the development of gastric cancer by inhibiting the *TNFSF15* expression. However, Kakuta et al. reported that the reporter construct with TNFSF15 −358C allele showed higher transcription activity than that with −358T allele in stimulated Jurkat cells. However, in the aforementioned study, no difference was observed in the luciferase activity in U937 cells between pGL4 constructs with −358C and −358T alleles. These conflicting findings pointed to a possible different regulation of *TNFSF15* by various regulators in specific cell types [34].

Besides genetic variants, environmental factors also play an important role in the etiology of gastric cancer. Many epidemiological studies have shown that increased risk of gastric cancer was associated with *H. pylori* infection in gastric tissues [4], [35], [36]. In animal experiments, *H. pylori* infection has been demonstrated to result in gastric inflammation and eventually lead to gastric cancer in mice [37], [38]. It is known that tumor necrosis factors play important roles in the progression and severity of inflammation, thereby contributing to the development of *H. pylori*-induced gastric cancer [39]–[41]. For instance, levels of TNF-α, a pro-inflammatory cytokine, are elevated within *H. pylori* infected gastric mucosa [42]. More so, functional genetic variants of the *TNF-α* gene have been associated with the susceptibility to gastric cancer [43]. TNFSF15, as a member of the tumor necrosis factor superfamily, modulates cell growth and inflammation in many different cell types [13], [20], [28], [44]. In this study we have evaluated the influence of TNFSF15 and *H. pylori* infection on gastric cancer susceptibility and found there was a joint effect of −358C allele and *H. pylori* infection in raising the risk of gastric cancer. The subjects with *TNFSF15* −358CC genotype were at elevated risks for developing gastric adenocarcinoma compared with those with −358TT genotype in the *H. pylori* infected group, but not in the *H. pylori* negative group. These results implicate a synergistic interaction between *H. pylori*-induced inflammation and host genetic factors in the development of gastric cancer.
